# Persistent musculoskeletal pain and its association with future mental distress in adolescents: The Fit Futures study

**DOI:** 10.1186/s12889-025-25272-w

**Published:** 2025-12-07

**Authors:** Kaja Smedbråten, Kirsti Riiser, Morten Andersen, Britt Elin Øiestad

**Affiliations:** 1 Department of Rehabilitation Science and Health Technology, Faculty of Health Sciences, Oslo Metropolitan University, P.O. Box 4, St. Olavs Plass, Oslo, 0130 Norway; 2 Department of Child and Adolescent Health Promotion Services, Norwegian Institute of Public Health, Levanger, Norway

**Keywords:** Mental distress, Musculoskeletal pain, Adolescents, Cohort study

## Abstract

**Objective:**

The aim of this study was to investigate whether persistent musculoskeletal pain was associated with mental distress two years later in adolescents.

**Methods:**

Data for this prospective cohort study on Norwegian adolescents were obtained from the population-based Fit Futures study. A total of 535 students in first year of upper-secondary school, without mental distress at baseline, were included. The primary outcome was *mental distress* at the two-year follow-up, measured with the Hopkins Symptom Checklist–10. The exposure *persistent musculoskeletal pain* was defined as pain that persisted or recurred at least weekly for ≥ 3 months, in one or more musculoskeletal sites. *Moderate to severe persistent musculoskeletal pain* was defined as persistent musculoskeletal pain with an intensity of ≥ 5 on a numeric rating scale from 0 (no pain) to 10 (worst pain imaginable). The associations between the two exposures and mental distress were separately assessed with logistic regression analysis, adjusted for gender, parents´ education, chronic diseases, sleep duration and social acceptance among peers.

**Results:**

Adjusted for potential confounders, adolescents who reported persistent musculoskeletal pain at baseline had an odds ratio (OR) for mental distress at two-year follow up of 2.57 (95% CI 1.40–4.69) compared to those without persistent musculoskeletal pain at baseline. Adolescents who reported *moderate to severe* persistent musculoskeletal pain at baseline had an OR for mental distress of 4.92 (95% CI 2.37–10.22) compared to those without moderate to severe persistent musculoskeletal pain at baseline.

**Conclusion:**

In adolescents, persistent musculoskeletal pain was associated with mental distress two years later. Adolescents with moderate to severe persistent musculoskeletal pain were particularly vulnerable to developing mental distress. The findings from this study should be replicated in larger studies, and the mechanisms underlying the association further explored to strengthen the foundation for developing preventive strategies for mental distress in adolescents.

**Supplementary Information:**

The online version contains supplementary material available at 10.1186/s12889-025-25272-w.

## Introduction

Adolescence and young adulthood are usually considered to be the healthiest time of life [1], however, the last couple of decades a worrying high prevalence of mental disorders and chronic pain has been reported [2–5]. Mental disorders affect approximately one in eight 10-14-year-olds and nearly one in seven 15–19-year-olds, according to data from the Global Burden of Disease study [3] , and are a major contributor to disability among young people worldwide [6]. Depression, anxiety and behavioral disorders are common mental disorders in young people [3]. Besides diagnosed mental disorders, the prevalence of mental distress - often assessed through self-reported symptoms of depression and anxiety - has increased notably over the last decades, particularly among girls [7, 8]. Existing literature indicates that risk factors for developing mental distress and disorders in adolescents encompass a range of biological, psychological and social factors, including factors such as female sex, low socioeconomic status, problems in parental or peer relationships, sleep problems and traumatic experiences [9–11]. The large changes in brain development, identity formation and social dynamics [1, 12], alongside the peak of mental health problems that occur during adolescence and young adulthood [13], make this period crucial for preventive strategies. Enhancing our understanding of the factors associated with the development of mental health problems in young people is essential for effective prevention and treatment. Prioritizing these efforts can help prevent the persistence of mental disorders and distress into adulthood, thereby improving young individuals’ opportunities to lead healthy, normal, and active lives.

Another important contributor to disability in young people is musculoskeletal pain [6]. International data show that approximately one in four children and adolescents report persistent or recurrent musculoskeletal pain that lasts for at least 3 months [2]. In Norway, one in three adolescents reported pain the past month in a large population-based study [14]. Adolescents with persistent pain report reduced health-related quality of life [15, 16], alongside limitations in ability to participate in daily activities [17], loneliness [18] and high levels of school absenteeism [19].

Importantly, musculoskeletal pain and mental distress often co-occur [20, 21], and individuals with co-occurrent complaints tend to experience higher levels of disability [22, 23]. The relationship between these two conditions is complex and not yet fully understood. Their association may either result from one condition causing the other, from a dynamic where they mutually reinforce each other, and/or from a shared predisposition that increases vulnerability to both [24]. Increasing our understanding of the temporal relationship between these conditions could contribute to the groundwork of preventive interventions for adolescents and help identify high-risk groups at an earlier stage. Research has reported that mental distress might be an early risk factor for musculoskeletal pain [25, 26]. There is less research on musculoskeletal pain as a potential risk factor for developing mental health problems in young people. Among previous studies, some have assessed pain in general, not emphasising musculoskeletal pain specifically [27–31], or examined the association within a clinical sample of adolescents with chronic pain and not a population-based sample [32]. Anyhow, a Norwegian population-based study identified an association between multisite musculoskeletal pain at age 15–16 and anxiety disorder in young adulthood [33]. Another Norwegian study investigating various musculoskeletal pain sites at age 15–16 found an association with mental distress three years later, with statistically significant results for most pain sites [34]. A Finnish study found that adolescents who reported multisite musculoskeletal pain at both age 16 and age 18 had an increased risk of experiencing mental distress at age 18 [35]. Conversely, another study that assessed persistent back and neck pain in adolescents, found no significant association with subsequent mental disorders [36]. Although the latter study examined persistent pain, it did not account for the severity of back/neck pain in its analysis. One of the Norwegian studies that explored a measure related to severity, by examining the number of pain sites, found higher odds ratios for developing mental distress for adolescents reporting 3–5 than 1–2 pain sites [34]. Even though experiencing pain is common in adolescence, it might not be a disturbing problem unless the pain becomes severe and interferes with daily life. Pain is a sensory and emotional experience, thus including severeness in the assessment of pain might be essential to identify a particularly vulnerable group.

The sparse and somewhat conflicting evidence on the relationship between musculoskeletal pain and mental distress in adolescents underscores the need for further research in this area. Investigating whether the inclusion of pain severity in musculoskeletal pain assessments can help identify particularly vulnerable groups at risk of developing mental distress, is an important avenue to explore. In this study, we aimed to investigate whether persistent musculoskeletal pain was associated with mental distress two years later in adolescents. Our hypotheses were (1) that persistent musculoskeletal pain is associated with mental distress two years later, and (2) that adolescents with moderate to severe persistent musculoskeletal pain are particularly at risk of developing mental distress.

## Methods

### Study design, participants and settings

**Fig. 1:**
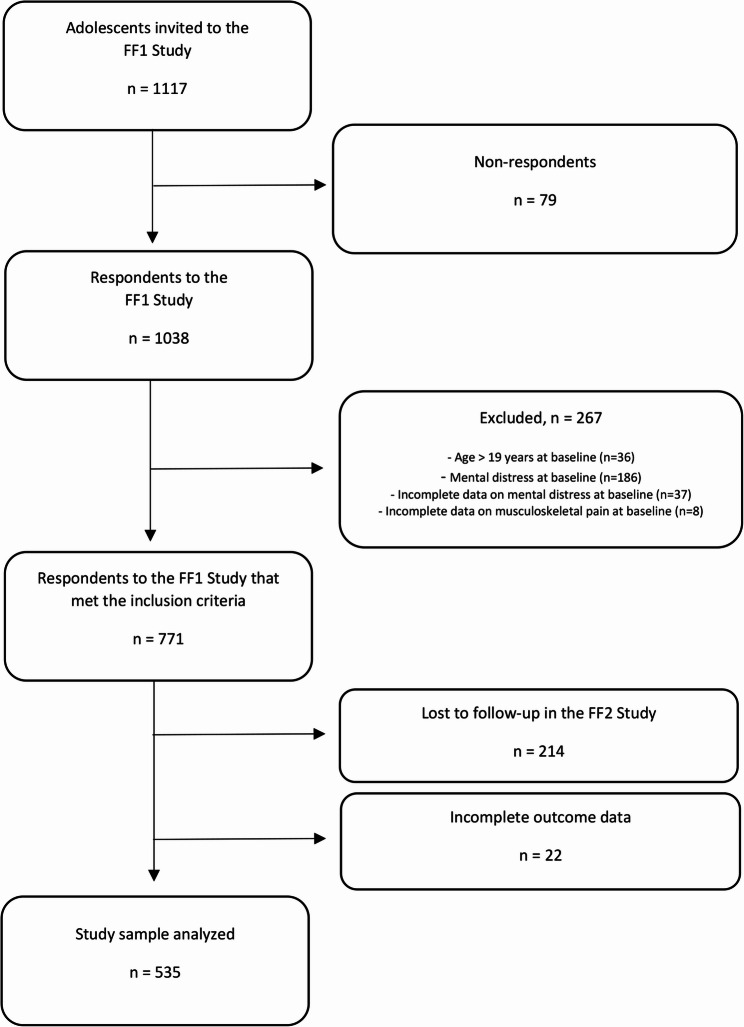
Flowchart of the study sample

This is a prospective cohort study on Norwegian adolescents, using data from the population-based Fit Futures study. The first wave of the study, Fit Futures 1 (FF1), was conducted in 2010–2011, inviting all first-year students from upper-secondary schools in the municipalities of Tromsø and Balsfjord, Northern Norway. The second wave, Fit Futures 2 (FF2), took place two years later (2012–2013), and included all FF1 participants as well as other third-year students attending the same schools. Our sample consists of participants who took part in both FF1 and FF2. Of 1117 students invited to FF1, 1038 participated (92.9%). Students above 19 years of age at baseline were excluded from our sample (*N* = 36). Moreover, to focus on new (incident) cases of mental distress, 186 students were excluded because they reported mental distress at baseline, and 37 because of incomplete data on baseline mental distress. Eight participants were excluded due to incomplete data on musculoskeletal pain at baseline. Of 771 respondents in FF1 that met our inclusion criteria, 557 (72.2%) participated in FF2 and 535 (69.4%) had complete outcome data (Fig. 1).

The data collection consisted of several examinations, including a clinical interview and an electronic questionnaire, with questions on health, lifestyle and social factors (Supplementary material 1). Data collection took place during school hours at a research unit at the University Hospital of North Norway. Additional details about the study have been published previously [37, 38].

All participants provided written informed consent, and participants younger than age 16 also provided written informed consent from a guardian. The current study was approved by the Regional Committees for Medical and Health Research Ethics in Norway (Reference number 259549) and the Norwegian Agency for Shared Services in Education and Research (formerly Norwegian Centre for Research Data) (Reference number 282753). Reporting of this study follows the Strengthening the Reporting of Observational Studies in Epidemiology (STROBE) statement [39].

### Outcome

The primary outcome was symptoms of mental distress, hereafter referred to as “mental distress”, measured at the two-year follow-up using the Hopkins Symptom Checklist – 10 (HSCL-10) [40, 41]. The questionnaire measures mainly symptoms of depression and anxiety and consists of 10 items where participants are asked if they have been bothered with the following feelings during the last week: “*suddenly scared for no reason”*,* “feeling afraid or worried”*,* “faintness or dizziness”*,* “feeling tense or keyed up”*,* “blaming yourself for things”*,* “sleep difficulties”*,* “depression or sadness”*,* “feeling useless or worthless”*,* “feeling that everything is an effort”*, and/or *“feeling hopeless about the future”.* Each item is rated on a four-point scale ranging from 1 (not at all bothered) to 4 (extremely bothered), and a mean score (1–4) was calculated. A cut-off for symptoms of mental distress was set as a mean score of >1.85 [40]. The questionnaire has demonstrated both validity [42], as well as acceptable reliability [43], in studies conducted among Norwegian adolescents. Mental distress was measured with the same questionnaire at baseline to identify our sample of adolescents without mental distress.

### Exposure

*Persistent musculoskeletal pain* was assessed with the following questions: “Do you have persistent or recurring pain that has lasted for 3 months or more?”(yes/no), [if yes] “how often do you usually experience pain?” and “where does it hurts?”. The last question was followed by a list of pre-specified body regions. Persistent musculoskeletal pain was defined as pain that persisted or recurred at least weekly for 3 months or more, in one or several of the following pain sites: “shoulder”, “arm/elbow”, “hand”, “hip”, “thigh/knee/shin”, “ankle/foot”, “jaw/temporomandibular joint”, “neck”, “upper back”, “lower back” and/or “chest” [44]. The questionnaire was developed for the Fit Futures study, and it is not formally validated.

*Moderate to severe persistent musculoskeletal pain* was defined as persistent musculoskeletal pain with a pain intensity of ≥ 5 on a numeric rating scale from 0 (no pain) to 10 (worst pain imaginable) [45]. Hence, adolescents who reported both persistent musculoskeletal pain *and* a pain intensity of five or more were assumed to have moderate to severe persistent musculoskeletal pain.

### Potential confounding factors

Gender [9, 46], socioeconomic status [25, 47], chronic diseases [9, 48], sleep [49, 50] and social network (e.g. peer relationships, social acceptance) [10, 51] were considered as potential confounding factors based on previous empirical findings and available data. *Gender* was measured as girls/boys. *Socioeconomic status* was assessed by parental educational level and categorised by the highest completed education by mother or father: “at least one parent with higher education”, “primary/secondary school” and “don´t know”. *Chronic diseases* were assessed with the question “do you have any chronic or persistent diseases?”, and the responses were categorised as yes/no. *Sleep duration* was assessed with the question “how many hours of sleep do you normally get per night?”. The responses were categorised as < 8 h and ≥ 8 h of sleep, where 8 h corresponds to the minimum recommended sleep duration for teenagers [52]. *Social acceptance among peers* was used as a potential confounding factor, assessed with five items from the revised Norwegian version of Harter´s Self-perception Profile for Adolescents [53, 54], concerning perceived ease of making friends and social acceptance by peers. The items were rated on a four-point scale. Negative worded questions were reversed, and an average score was calculated (1–4). A higher score indicates higher perceived social acceptance among peers.

### Other descriptive variables

*Age*, and an overview of *pain location* (“neck/shoulder pain”, “back pain”, “lower extremity pain”, “hand/arm pain”, “chest pain” and “jaw pain”) have been presented to describe the sample.

### Statistical analyses

Analyses were performed using STATA statistical software system, version 18 [55]. Characteristics of the study sample were described with counts and percentages for categorical variables and with median and interquartile range (IQR) for continuous variables (due to skewed distributions). Possible differences between respondents and nonrespondents at follow-up were assessed with Mann-Whitney U test (continuous data with a skewed distribution) and Chi-Square tests (categorical data).

A two-year incidence rate of new episodes of mental distress was calculated by dividing the number of participants with mental distress at follow-up by the total number of participants in the study sample, and it was reported as a percentage.

The association of persistent musculoskeletal pain and moderate to severe persistent musculoskeletal pain at baseline with mental distress at 2-year follow-up were assessed with logistic regression analysis. Separate models were built for each exposure. The results were expressed by crude and adjusted odds ratios (ORs) with 95% confidence intervals (CI). Adjustments were conducted in two blocks: firstly, for gender and parents’ educational level (model 1), and secondly for gender, parents´ educational level, chronic diseases, sleep duration and social acceptance among peers (model 2). There was generally little missing data in exposures and confounders (up to 2%), therefore, complete case analyses were conducted. *P*-values < 0.05 were considered statistically significant.

## Results

### Sample characteristics

Baseline characteristics of the study sample are shown in Table 1. Median age at baseline was 16 years (IQR 0), and 51.8% of the participants were girls. A total of 74 (13.8%) reported persistent musculoskeletal pain and 38 (7.1%) reported moderate to severe persistent musculoskeletal pain at baseline. Among musculoskeletal pain sites, pain in the lower extremities was the most commonly reported, followed by back pain. The proportion of girls was 58.1% and 65.8% in the groups of adolescents with persistent musculoskeletal pain and moderate to severe persistent musculoskeletal pain, respectively, and 50.8% in the group without persistent musculoskeletal pain. The proportion of adolescents who had one or more chronic diseases was 39.2% and 42.1% in the groups of adolescents with persistent musculoskeletal pain and moderate to severe persistent musculoskeletal pain, respectively, whereas 25.3% in the group without persistent musculoskeletal pain. Also, the proportion of adolescents reporting less than 8 h of sleep per day was 75.7% among those with persistent musculoskeletal pain, 78.9% among those with moderate to severe persistent musculoskeletal pain, and 72.1% among those without persistent musculoskeletal pain. About half of the adolescents without persistent musculoskeletal pain (52.7%) reported that they had at least one parent with higher education, while nearly one fourth (23.7%) did not know the educational level of their parents. Among adolescents with persistent musculoskeletal pain and moderate to severe persistent musculoskeletal pain, 51.4% and 42.1% reported that they had at least one parent with higher education, respectively.

**Table 1:** Baseline characteristics of the study sample

Characteristics	No persistent musculoskeletal pain (*n* = 461)	Persistent musculoskeletal pain ^a^ (*n* = 74)	Moderate to severe persistent musculoskeletal pain ^b^ (*n* = 38)	Overall (*n* = 535)	Missing *n* (%)
Gender, girls, *n* (valid %)	234 (50.8)	43 (58.1)	25 (65.8)	277 (51.8)	
Parents` education, *n* (valid %)					
Higher education (mother or father)	240 (52.7)	38 (51.4)	16 (42.1)	278 (52.6)	6 (1.1)
Primary/secondary school	107 (23.5)	15 (20.3)	11 (28.9)	122 (23.1)	
Don’t know	108 (23.7)	21 (28.4)	11 (28.9)	129 (24.4)	
Chronic diseases, yes, *n* (valid %)	116 (25.3)	29 (39.2)	16 (42.1)	145 (27.3)	3 (0.6)
Social acceptance among peers ^c^, median (IQR)	3.4 (0.4)	3.4 (0.6)	3.4 (0.6)	3.4 (0.5)	11 (2.0)
Sleep duration, *n* (valid %)					
< 8 h	330 (72.1)	56 (75.7)	30 (78.9)	386 (72.6)	3 (0.6)
≥ 8 h	128 (27.9)	18 (24.3)	8 (21.1)	146 (27.4)	
Pain location ^d^, *n* (valid %)	-				
Neck and/or shoulder		31 (41.9)	16 (42.1)	31 (5.8)	
Back		39 (52.7)	19 (50.0)	39 (7.3)	
Lower extremity		43 (58.1)	21 (55.3)	43 (8.0)	
Arm/hand		12 (16.2)	7 (18.4)	12 (2.2)	
Chest		9 (12.2)	6 (15.8)	9 (1.7)	
Jaw		4 (5.4)	4 (10.5)	4 (0.7)	

Valid %: the percentages are calculated by considering the valid responses only

^a^ Weekly musculoskeletal pain for three months or more

^b^ Weekly musculoskeletal pain for three months or more with an intensity of 5 or more on a Numeric Rating Scale (0–10) (the participants in this group are also included in the group of individuals with persistent musculoskeletal pain)

^c^ Social acceptance among peers (1–4), higher values indicate higher social acceptance

^d^ The pain location groups are not mutually exclusive, as participants can have reported pain from several locations

Compared to the group of participants who completed the follow-up study, the group of individuals who were lost to follow-up or had incomplete outcome data had a lower proportion of girls. No other statistically significant differences were found between these two groups (Supplementary material 2).

### Two-year incidence of mental distress

The two-year incidence of mental distress was 17.4% (95% CI 14.3–20.9) (*n* = 93).

### Persistent musculoskeletal pain at baseline and mental distress at two-year follow-up

In the crude analysis, adolescents who reported persistent musculoskeletal pain at baseline had an OR of 2.14 (95% CI 1.22–3.76) for mental distress at two-year follow-up, compared to adolescents without persistent musculoskeletal pain (Table 2). Adjusted for potential confounders, the OR for mental distress at two-year follow up was 2.57 (95% CI 1.40–4.69) for adolescents with persistent musculoskeletal pain at baseline.

**Table 2:** Logistic regression analyses of the association between persistent musculoskeletal pain at baseline and mental distress at the two-year follow-up ^a^

	Crude OR (95% CI)	Adjusted 1 ^d^ OR (95% CI)	Adjusted 2 ^e^ OR (95% CI)
Persistent musculoskeletal pain ^b^	2.14 (1.22–3.76)	2.08 (1.17–3.70)	2.57 (1.40–4.69)
Moderate to severe persistent musculoskeletal pain ^c^	4.48 (2.26–8.89)	4.10 (2.04–8.23)	4.92 (2.37–10.22)

*Abbreviations*: *OR* Odds ratio, *CI* Confidence interval

“Persistent musculoskeletal pain” and “Moderate to severe persistent musculoskeletal pain” were investigated in separate analyses

^a^ Measured by the Hopkins Symptom Checklist–10 [41] (1–4), > 1.85 indicating mental distress [40]

^b^ Weekly musculoskeletal pain for three months or more

^c^ Weekly musculoskeletal pain for three months or more with a pain intensity of at least 5/10

^d^ Adjusted for gender and parents` educational level

^e^ Adjusted for gender, parents` educational level, chronic diseases, sleep duration and social acceptance among peers

Adolescents who reported *moderate to severe* persistent musculoskeletal pain at baseline had an OR of 4.48 (95% CI 2.26–8.89) for mental distress at two-year follow-up, compared to adolescents without moderate to severe persistent musculoskeletal pain in the crude analysis (Table 2). Adjusted for potential confounders, the OR for mental distress at two-year follow-up was 4.92 (95%CI 2.37–10.22) for adolescents with moderate to severe persistent musculoskeletal pain at baseline.

## Discussion

In this population-based prospective cohort study, persistent musculoskeletal pain, particularly moderate to severe persistent musculoskeletal pain, was associated with mental distress two years later in adolescents without mental distress at baseline.

Our findings are in accordance with a Norwegian three-year follow-up study with more than 2500 study participants without mental distress at baseline, reporting an association between pain in 15/16-year-olds and mental distress at age 18/19 years [34]. In this previous study, musculoskeletal pain was measured as neck/shoulder, arm/leg/knee or back pain experienced at least once over the past 12 months, and an association was found for most pain sites, except for arm/leg/knee pain in boys [34]. To our knowledge, no other larger longitudinal studies have investigated musculoskeletal pain on future mental distress among adolescents without mental distress at baseline. However, a study using data from the Northern Finland Birth Cohort 1986, with no data on mental distress at baseline, supported our findings by showing that persistent multisite musculoskeletal pain from age 16 to age 18 was associated with mental distress at age 18 in both boys and girls [35]. Moreover, a Norwegian cohort study, which did not exclude participants with mental disorders at baseline, found that multisite musculoskeletal pain in adolescence was associated with mental health disorders in young adulthood [33]. However, in that study, the association remained significant for anxiety disorders but not for mood disorders after adjusting for psychosocial and mental health problems at baseline. The authors speculated that there might have been mediating or confounding effects indicating an intertwined association between musculoskeletal pain and psychosocial problems in predicting mental health disorders in young adulthood [33].

A comprehensive understanding of the underlying mechanisms linking persistent pain to future mental distress in young people remains uncertain. Adolescence is a crucial phase of life characterised by the development of independence from parents and the formation of social and emotional habits and skills [1, 12], all of which are important for physical and mental well-being. Living with pain might lead to social isolation, as these individuals might miss out on sports and other spare time activities [17, 56], and experience increased absence from school [19]. This potential isolation or impact on social life may place adolescents at risk of mental health problems, including mental distress [57], and could potentially explain the observed association with persistent musculoskeletal pain. The strong association between moderate to severe persistent musculoskeletal pain and subsequent mental distress observed in this study might supports this theory, as individuals experiencing more severe pain might suffer the greatest impact on their social lives [58]. Nevertheless, the association between persistent musculoskeletal pain and subsequent mental distress might also be explained by shared underlying mechanisms [59, 60]. The shared vulnerability model describes potential mechanisms that may explain the comorbidity of mental health problems and pain [24]. According to this model, shared biological and psychological vulnerability factors - such as heightened anxiety sensitivity and a low threshold for sympathetic arousal – may give rise to negative emotional responses (e.g. fear, anxiety) in relation to stressful life events (e.g. traumatic incident, injury) [24, 59]. In turn, maintaining factors such as avoidance behaviour, negative cognitions and/or hypervigilance, which can emerge in response to the emotional reaction, may further contribute to the development of both mental health problems and persistent pain [24, 59]. Previous cross-sectional studies have demonstrated a high co-occurrence of pain and mental distress in young people [20, 21]. Furthermore, the observed association between musculoskeletal pain and mental distress in both the current and some previous longitudinal studies [34, 35], along with evidence of a possible reverse relationship [25], suggests a bidirectional connection, which may support the theory of shared underlying mechanisms. However, a prior study on the same cohort of adolescents as this study, found no association between mental distress at baseline and persistent musculoskeletal pain two years later [44]. Also, a longitudinal study investigating the potential reciprocal relationship between pain and mental health among adolescents with chronic pain or depression, respectively, found that changes in pain intensity were predictive of subsequent depressive symptoms, while changes in depressive symptoms had less impact on subsequent pain [32]. After all, combining the theory of shared cognitive and emotional mechanisms with the inherently vulnerable and influenceable state of young people, could potentially lead to a plausible explanation for the development of mental distress in adolescents with persistent pain observed in the current study. The mechanisms underlying this relationship should, however, be further explored.

### Strengths and limitations

One strength of this study was the cohort study design, which enabled the examination of the temporal relationship between persistent musculoskeletal pain and mental distress. Additionally, by excluding individuals with mental distress at baseline, we reduced the potential for reverse causation. Nevertheless, the study has some limitations. Almost one-third of the sample was lost to follow-up or had incomplete outcome data. Analyses comparing baseline values for those lost and those who remained in the study showed that the group who was lost had a lower proportion of girls. However, no other statistically significant differences between the groups were observed. Although we excluded individuals with mental distress at baseline, symptoms of mental distress can be fluctuating, particularly when measured as experiences “during the last week”. As this short time frame may not have fully captured the participants’ overall mental health status, some cases of mental distress observed at follow-up may reflect ongoing or previously undetected symptoms rather than incident cases. Furthermore, as adolescent pain - like mental distress - can fluctuate over time, this may have affected the validity of the temporal association between pain and distress. To better capture the dynamics of these variables over time, future research should aim to include repeated measures of both pain and distress. Additionally, the questionnaire used to assess musculoskeletal pain was not formally validated. Future studies should aim to use validated pain assessment tools with known psychometric properties to ensure reliable and accurate measurement of musculoskeletal pain in adolescents. Another limitation was the small number of exposed cases with mental distress at follow-up, and the wide CIs, leading to imprecise estimates. Nonetheless, the results, especially for moderate to severe musculoskeletal pain, suggested strong point estimates. While we adjusted for several potential confounding factors, we were unable to account for life events, such as past traumatic experiences, due to a lack of data. We also acknowledge the potential influence of other unmeasured confounding factors, including temperamental factors. Furthermore, we adjusted for socioeconomic status based on parental education, however, about one-fourth of the adolescents did not know their parents’ educational level. Given the described limitations, the findings of this study should be interpreted with caution and replicated in future studies that address the identified methodological concerns.

### Implications

Health care providers should be aware that persistent musculoskeletal pain, particularly moderate to severe persistent musculoskeletal pain, is associated with an increased odds of future mental distress in adolescents. Even though we do not know the causal relationship between these conditions, they tend to either co-occur or be strongly associated. Thus, one relevant measure for clinicians is to include assessment of mental distress when consulting adolescents with persistent pain conditions seeking care. Moreover, the observed association between persistent musculoskeletal pain and subsequent mental distress might suggest that addressing such pain early, including targeting potential shared underlying factors, can be instrumental in preventing the development of mental distress. For example, school-based interventions could play a key role in such efforts, as schools provide an accessible and structured setting for promoting early identification, health education and intervention. However, the findings from this study should be replicated in larger studies, which also include a broader range of variables, to capture the complexity of the studied phenomenon and to facilitate exploration of potential mechanisms underlying the observed association. Understanding these mechanisms is essential for laying the groundwork for the development of effective preventive interventions. Preventing mental distress in adolescence is crucial, as these young individuals are on the verge of entering adulthood, gaining independence from parents, and making important life decisions regarding education, relationships, and careers.

## Conclusion

In adolescents, persistent musculoskeletal pain was associated with mental distress two years later. Adolescents with moderate to severe persistent musculoskeletal pain were particularly vulnerable to developing mental distress. The findings from this study should be replicated in larger studies, and the mechanisms underlying the association further explored to strengthen the foundation for developing preventive strategies for mental distress in adolescents.

## Supplementary Information

Supplementary Material 1.

Supplementary Material 2.

## Data Availability

The data underlying the results presented in this study were used under license for the current study and cannot be shared publicly due to restrictions imposed by the Regional Committees for Medical and Health Research Ethics (post@helseforskning.etikkom.no), in accordance with Norwegian law. However, these data can be made available upon reasonable request to the Fit Futures Study (fitfutures@uit.no), provided the request complies with ethical and legal requirements.

## Abbreviations

CI: Confidence interval
FF1: Fit Futures 1
FF2: Fit Futures 2
OR: Odds ratio
STROBE: Strengthening the Reporting of Observational Studies in Epidemiology
WHO: World Health Organization
