# Supplementary material for: Persistent musculoskeletal pain and its association with future mental distress in adolescents: The Fit Futures study
Source: BMC Public Health. 2025 Dec 7;26:158. doi: 10.1186/s12889-025-25272-w (PMC12797425; doi:10.1186/s12889-025-25272-w)
Supplement: Supplementary file 1 — Supplementary Material 1. [file 12889_2025_25272_MOESM1_ESM.docx]

**Overview of questions used in this study.**Contains questions and response alternatives, translated from Norwegian to English

**Are you**

- Girl
- Boy

**Do you have any chronic or persistent disease?**

- Yes
- No

**What is the highest education completed by your mother?**

- Primary school, 9 years
- Occupational high school
- High school
- College less than 4 years
- College

**What is the highest education completed by your father?**

- Primary school, 9 years
- Occupational high school
- High school
- College less than 4 years
- College

**Below are some questions about how you perceive yourself**
Check the option that best fits you.

(Five items from the revised Norwegian version of Harter´s Self-perception Profile for Adolescents [1, 2])

|  | Very true | Somewhat true | Somewhat untrue | Very untrue |
| --- | --- | --- | --- | --- |
| I find it quite difficult to make friends  I have many friends  My peers don’t like me  I am popular among my peers  I feel accepted by my peers |  |  |  |  |

**Here is a list of various problems. Have you experienced any of these during the past week (including today)?**

(Hopkins Symptom Checklist – 10 (HSCL-10) [3, 4])

|  | Not at all bothered | Slightly bothered | Quite a bit bothered | Extremely bothered |
| --- | --- | --- | --- | --- |
| Suddenly scared for no reason  Feeling afraid or worried  Faintness or dizziness  Feeling tense or keyed up  Blaming yourself for things  Sleep difficulties  Depression, sadness  Feeling useless or worthless  Feeling that everything is an effort  Feeling hopeless about the future |  |  |  |  |

**How many hours do you usually sleep per night?**

- 4 hours or less
- 4 ½ hour
- 5 hours
- 5 ½ hour
- 6 hours
- 6 ½ hour
- 7 hours
- 7 ½ hour
- 8 hours
- 8 ½ hour
- 9 hours
- 9 ½ hour
- 10 hours
- 10 ½ hour
- 11 hours
- 11 ½ hour
- 12 hours or more

**Do you have persistent or recurring pain that has lasted for 3 months or more?**

- Yes
- No

**How often do you have pain?**

- All the time, without interruption
- Every day, but not all the time
- Every week, but not every day
- Less often than every week

**Where does it hurt?**

| - Shoulder - Arm/elbow - Hand - Hip - Thigh/ knee/shin - Ankle/foot - Head/face - Jaw/temporomandibular joint - Neck - Upper back - Lower back - Chest - Abdomen - Genital/ genitals area | Left side | Right side |
| --- | --- | --- |
|  | The middle | |

**How severe would you say your pain usually is?**
Answer on a scale from 0 to 10, where 0 means no pain and 10 means the worst imaginable pain. If you experience multiple types of pain, answer based on the one that bothers you the most.

- 0
- 1
- 2
- 3
- 4
- 5
- 6
- 7
- 8
- 9
- 10

REFERENCES:

1. Wichstrøm L. Harter's Self-Perception Profile for Adolescents: reliability, validity, and evaluation of the question format. J Pers Assess. 1995;65(1):100-16.

2. Harter S. Manual for the Self-Perception Profile for Adolescents. Denver, CO: University of Denver Press; 1988.

3. Strand BH, Dalgard OS, Tambs K, Rognerud M. Measuring the mental health status of the Norwegian population: A comparison of the instruments SCL-25, SCL-10, SCL-5 and MHI-5 (SF-36). Nordic journal of psychiatry. 2003;57(2):113-8.

4. Derogatis LR, Lipman RS, Rickels K, Uhlenhuth EH, Covi L. The Hopkins Symptom Checklist (HSCL): A self-report symptom inventory. Behav Sci. 1974;19(1):1-15.
