# Supplementary material for: Persistent musculoskeletal pain and its association with future mental distress in adolescents: The Fit Futures study
Source: BMC Public Health. 2025 Dec 7;26:158. doi: 10.1186/s12889-025-25272-w (PMC12797425; doi:10.1186/s12889-025-25272-w)
Supplement: Supplementary file 2 — Supplementary Material 2. [file 12889_2025_25272_MOESM2_ESM.docx]

| **Comparison of baseline characteristics of respondents and non-respondents ^a^ of the follow-up study** | | | | |
| --- | --- | --- | --- | --- |
| **Characteristics** | **Respondents**  **(n = 535)** | **Non-respondents ^a^**  **(n= 236)** | **P-value** | |
| Age, years, median (IQR) | 16 (0) | 16 (0) | 0.43 | |
| Sex, girls, n (%) | 277 (51.8) | 60 (25.4) | < 0.01 | |
| Parents` education, n (valid %) |  |  | 0.08 | |
| Higher education (mother or father) | 278 (52.6) | 104 (45.0) |  | |
| Primary / secondary school | 122 (23.1) | 54 (23.4) |  | |
| Don’t know | 129 (24.4) | 73 (31.6) |  | |
| *Missing, n* | *6* | *5* |  | |
| Persistent musculoskeletal pain ^b^, yes,  n (valid %) | 74 (13.8) | 34 (14.4) | 0.83 | |
| Moderate/severe persistent musculoskeletal pain ^c^ , n (valid %) | 38 (7.1) | 17 (7.2) | 0.96 | |
| Chronic diseases, yes, n (valid %) | 145 (27.3) | 59 (25.1) | 0.54 | |
| *Missing, n* | *3* | *1* |  | |
| Social acceptance among peers ^d^,  median (IQR) | 3.4 (0.5) | 3.4 (0.6) | 0.48 | |
| *Missing, n* | *11* | *3* |  | |
| Sleep duration, n (valid %) |  |  | 0.18 | |
| < 8 hours | 386 (72.6) | 158 (67.8) |  | |
| ≥ 8 hours | 146 (27.4) | 75 (32.2) |  | |
| *Missing, n* | *3* | *3* |  | |
| ^a^  Non-respondents include both those who did not attend the follow-up study at all, and those who did not have valid responses on mental distress (the outcome) at follow-up  ^b^  Weekly musculoskeletal pain for three months or more  ^c^ Weekly musculoskeletal pain for three months or more with an intensity of 5 or more on a Numeric Rating Scale (0-10)  ^d^ Social acceptance among peers (1-4), higher values indicate higher social acceptance | | | |  |
